# Supplementary material for: Marine ecosystem shifts with deglacial sea-ice loss inferred from ancient DNA shotgun sequencing
Source: Nat Commun. 2023 Mar 24;14:1650. doi: 10.1038/s41467-023-36845-x (PMC10039020; doi:10.1038/s41467-023-36845-x)
Supplement: Supplementary file 1 — Supplementary Information [file 41467_2023_36845_MOESM1_ESM.pdf]

Supplementary Information

Marine ecosystem shifts with deglacial sea-ice loss inferred from ancient DNA shotgun sequencing

Heike H. Zimmermann, Kathleen R. Stoof-Leichsenring, Viktor Dinkel, Lars Harms, Luise Schulte, Marc-Thorsten Hütt, Dirk Nürnberg, Ralf Tiedemann, Ulrike Herzschuh

Supplementary note 1: Opening of cores and subsampling procedures to prevent contamination
Supplementary note 2: Processing of sequencing data
Supplementary note 3: Negative controls
Supplementary note 4: Damage patterns
Supplementary note 5: Signal validation of Salmonidae
Supplementary note 6: Test for temporal autocorrelation
Supplementary note 7: Correlation networks
Supplementary References

Supplementary note 1: Opening of cores and subsampling procedures to prevent contamination

During the expedition on RV Sonne cruise SO-201 "KALMAR" in 2009, the core liner was cut into 1 m sections on board of the ship, which is common practice. After measuring the magnetic susceptibility (not part of the manuscript) on the round-sections, each 1m-section was split into working and archive halves. The working halves were sampled onboard and completely used up, providing sample material, commonly in 1 cm-slices, for various working groups. Color reflectance measurements (not part of the manuscript) were made on the archive half sections, which were covered with transparent polystyrene foil before measurement. The archive halves were packed into plastic D-tubes and stored at ~4°C, within a few hours of recovery.

The sampling was carried out at GEOMAR, Kiel, in a sedimentology laboratory devoid of any molecular biology work. The lab is not used for experiments on modern phytoplankton, fish, or other living organisms. The surface of the table and the core liner were cleaned DNAExitus Plus which was rinsed off with MiliQ water. The plastic foil covering the sediment was removed sample-by-sample to limit exposure of the sediments to the surrounding air. Clean, single-use knives (soaked in DNAaway for 10 minutes, rinsed with 70% Ethanol and irradiated for 20 min on each side in a UV-crosslinker) were used to remove ~2mm of the exposed sediment. Then a sterile scalpel blade was used to remove a second layer of sediment (~2 mm). A sterile syringe, of which the front was cut off with a clean, single-use knive (treated as described above) directly before, was inserted aiming for a sample volume of up to 4 mL. The sample was transferred into a sterile 8 mL tube and stored at -20°C until DNA extraction. During the whole procedure, fullbody suits, exchangeable arm sleeves, face masks, and two pairs of gloves on top of each other were worn. The gloves were changed between each sample and additionally, when it was contaminated by sediment.

Supplementary note 2: Processing of sequencing data

On average 36,189,968 read pairs were retrieved per sample and 2,148,914 read pairs per negative control (Supplementary Table 1). The following analyses were carried out in R. We performed a PCoA using the *pcoa* function of the package ape[1] based on Bray-Curtis distances of the original dataset classified on family level and on the pelagic dataset after taxonomic filtering and resampling for comparison. The PCoA shows that the blanks have a distinct composition which is highly dissimilar from the samples (Supplementary Fig.2).

We checked completeness of pelagic and benthic taxa within the samples on family level with rarefaction curves. The rarefaction curves reflect that many taxa were cautiously filtered out to get stable signals over time (Supplementary Fig. 3). For our purposes however, reaching a saturation of the curves is not essential, because we are not investigating presence/absence of taxa, but rather semi-quantitative trends in ecosystem composition.

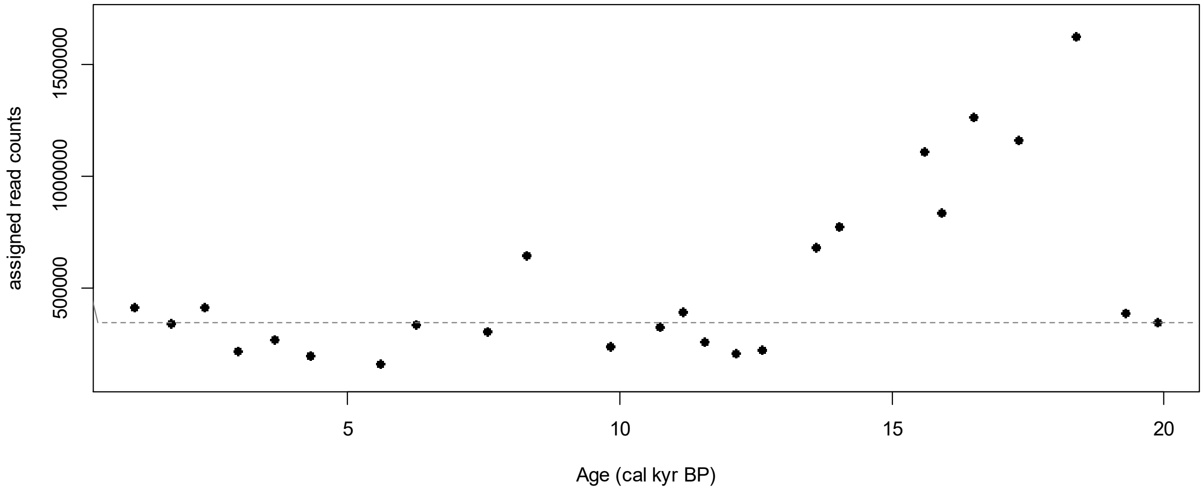

Supplementary Figure 1. Number of read counts per sample before taxonomic filtering. The median (324,044 counts) is indicated by the dashed line. The highest number of taxonomically assigned read counts can be found in samples dated between 18 and 13 cal kyr BP.

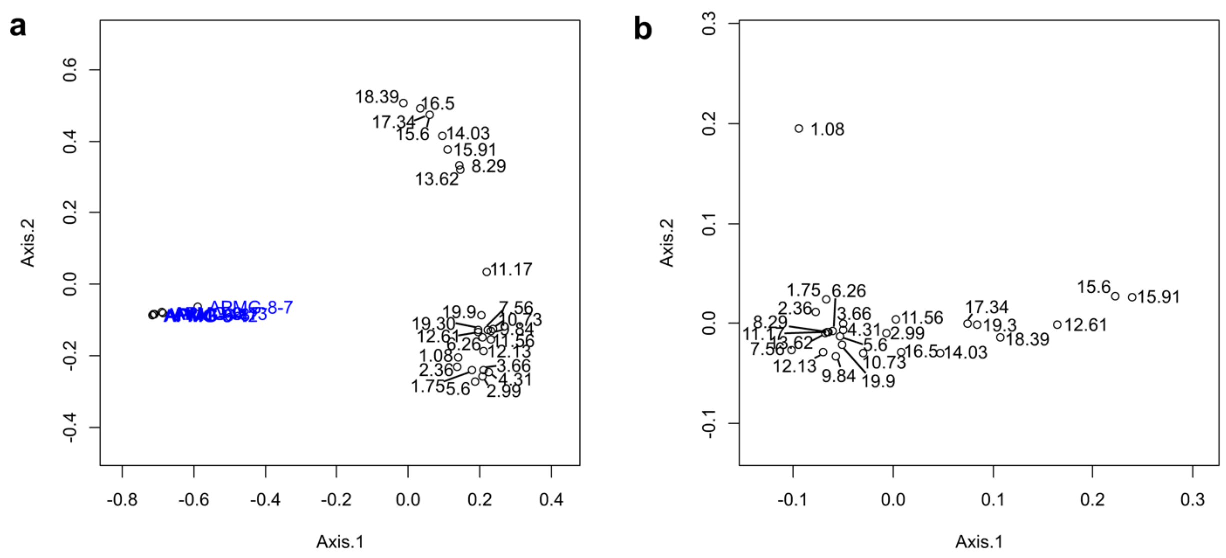

Supplementary Figure 2. PCoA based on Bray Curtis distances (a) of the original dataset classified on family level and (b) after filtering and resampling of pelagic taxa. Samples highlighted in blue are the blanks.

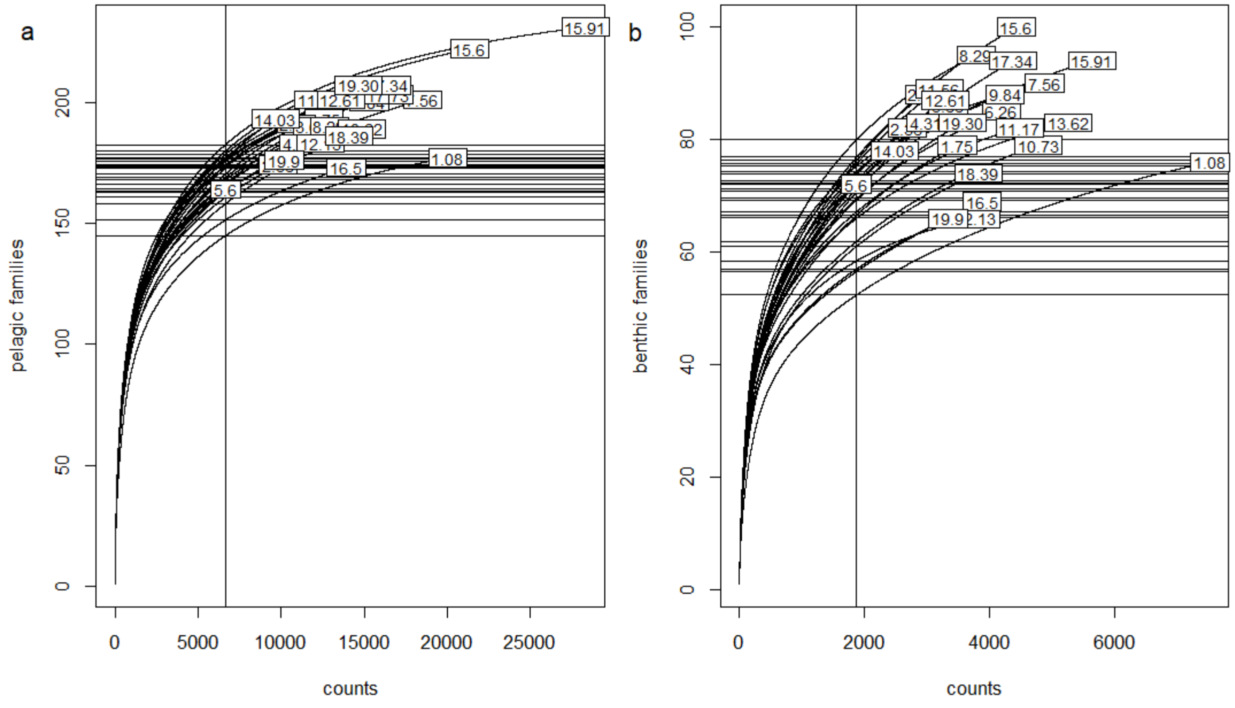

Supplementary Figure 3. Rarefaction curves for (a) pelagic and (b) benthic families before resampling. The vertical line indicates the lowest number of read counts for each of the datasets (pelagic = 6,593 counts; benthic = 1,839 counts) to which the two datasets were resampled, while horizontal lines mark the number of families detected for each sample after rarefaction.

Supplementary note 3: Negative controls

Throughout the library preparation process the negative controls were treated in the same way as the samples, also in terms of PCR cycle numbers for the indexing step. We retrieved between 395,384 and 2,494,694 sequences for the different negative controls of which between 0.01 and 30% were duplicates. After deduplication, trimming and filtering for low complexity, low quality, residual adapters and length only between 0.3 and 2.1% of the original read pairs were left. This suggests, our blanks contain very low levels of DNA and that increasing cycle numbers would predominantly amplify our duplication rate and increase the number of adapters in the sequencing data.

Of the 2,183 read pairs (merged and unmerged) 1,519 read pairs were retrieved on family level after filtering (Supplementary Table 2). The blanks are dominated, as expected, by bacterial- and human-derived sequences (Supplementary Fig. 4). The majority of taxa found in the blanks is however only represented by a single sequence count in total (Supplementary Fig. 5). The composition of the blanks is highly similar as shown in the PCoA plot (Supplementary Fig. 2) and markedly different from the samples.

Supplementary Table 1. Overview of assigned number of read counts and number of detected families in extraction and library negative controls.

Library name	Negative controls	Assigned read counts on family level	Number of families in blanks
APMG.6.7	Pooled extraction blanks (EH388, EH417, EH427, EH372, EH407)	235	35
APMG.6.8	Library blank 1	221	30
APMG.8.7	Pooled extraction blanks (EH437, EH457)	540	185
APMG.9.4	Library blank 2	187	25
APMG.9.12	Library blank 3	225	43
APMG.9.13	Library blank 4	111	23

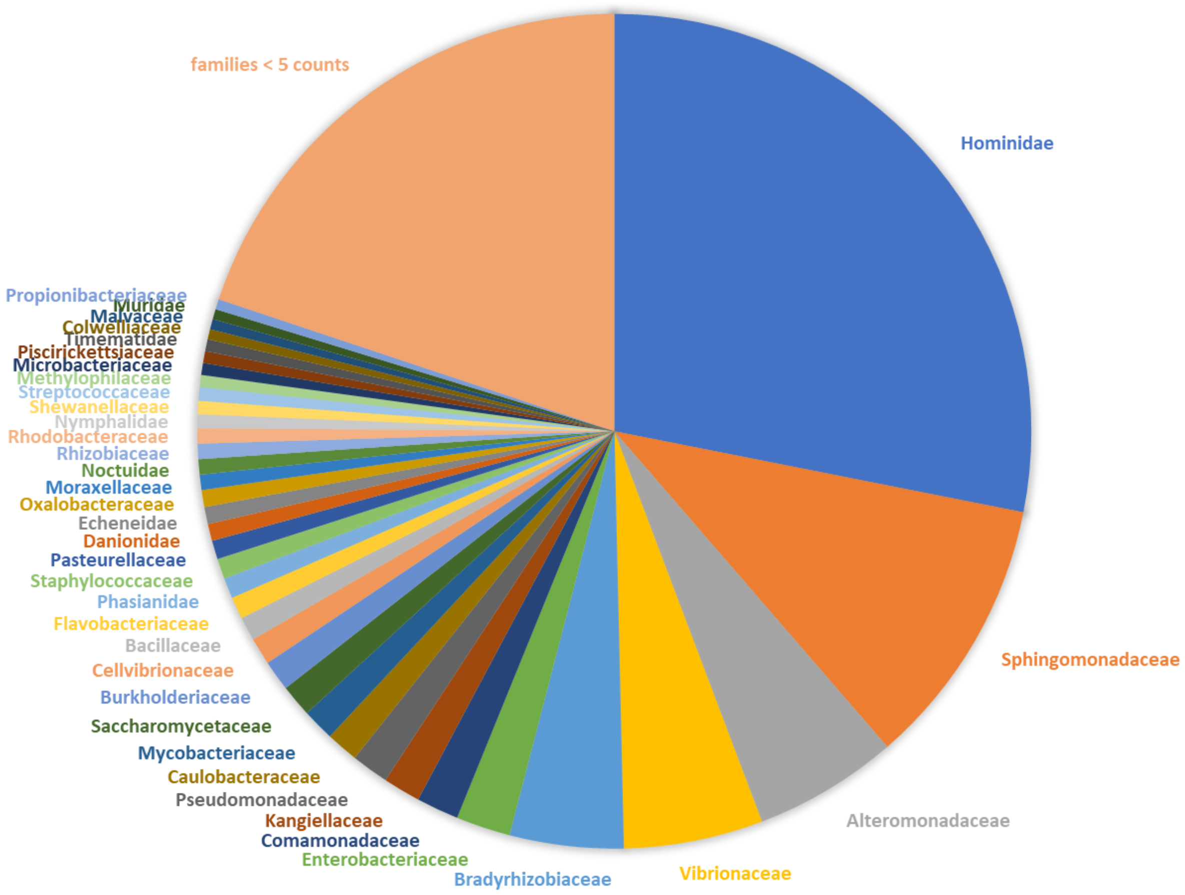

Supplementary Figure 4. The composition of all blanks combined shows that reads assigned to Hominidae and bacterial families are dominant in blanks. All taxa with less than five total read counts in blanks were grouped together.

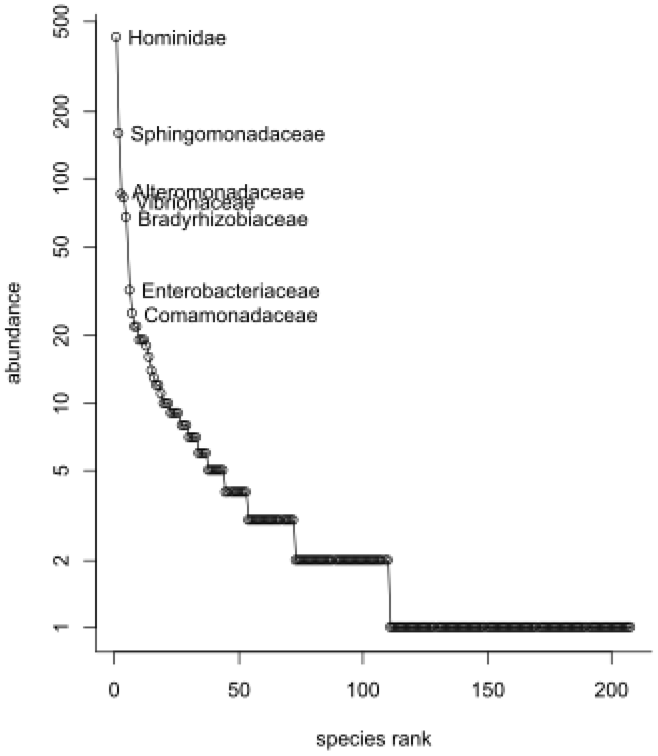

Supplementary Figure 5. Rank abundance curve of read counts per family in blanks. The 6 most abundant families are labeled, showing that highest levels of contamination were assigned to Hominidae and bacteria. The fish family Chanidae was removed from our analyses because of its high representation in blanks.

Supplementary note 4: Damage patterns

Damage patterns for pelagic and benthic focal taxa

We confirmed authenticity of pelagic and benthic focal taxa via damage pattern analysis using the automated HOPS pipeline[2]. The pipeline was run in full mode against the NCBI nt database (downloaded 03.11.2020) with options "filter" and "alignment" set to “ancient” and "1", respectively. The required index for the database was built using malt-build with default settings and the newest MEGAN6[3] mapping file (megan-nucl-map-Jul2020.db). The malt alignment of only merged reads against the full nt database was run for two benthic taxa (*Asteria rubens*, *Pecten maximus*), four eukaryotic algae (*Chaetoceros simplex*, *Thalassiosira pseudonana*, *Fragilariopsis cylindrus*, *Bathycoccus prasinos*), one prokaryotic algae (*Synechococcus*), three pelagic fish (*Oncorhynchus kisutch*, *Salmo trutta, Gadus morhua*), and for two taxa that we excluded from the data: 1. freshwater fish *Cyprinus carpio* (excluded because we assume a taxonomic mis-classification) and one dominant bacteria *Marinobacter* (excluded because we did not focus on non-phototrophic bacterial composition). The pipeline uses the MaltExtract function to identify the ratio of C>T (5’end) and G>A (3’end) substitutions for a selection of DNA reads (pre-classified as ancient) that have one mismatching lesion in their first 5 bases from either end. Damage pattern profiles of pre-selected ancient reads are provided for three time periods 1.08–5.6 cal kyr BP (Supplementary Fig. 6a,b), 6.3–12.6 cal kyr BP (Supplementary Fig. 6c,d), and 13.6–19.9 cal kyr BP (Supplementary Fig. 6e,f) and show increasing accumulation of damage over the three binned timeframes for pelagic and benthic families.

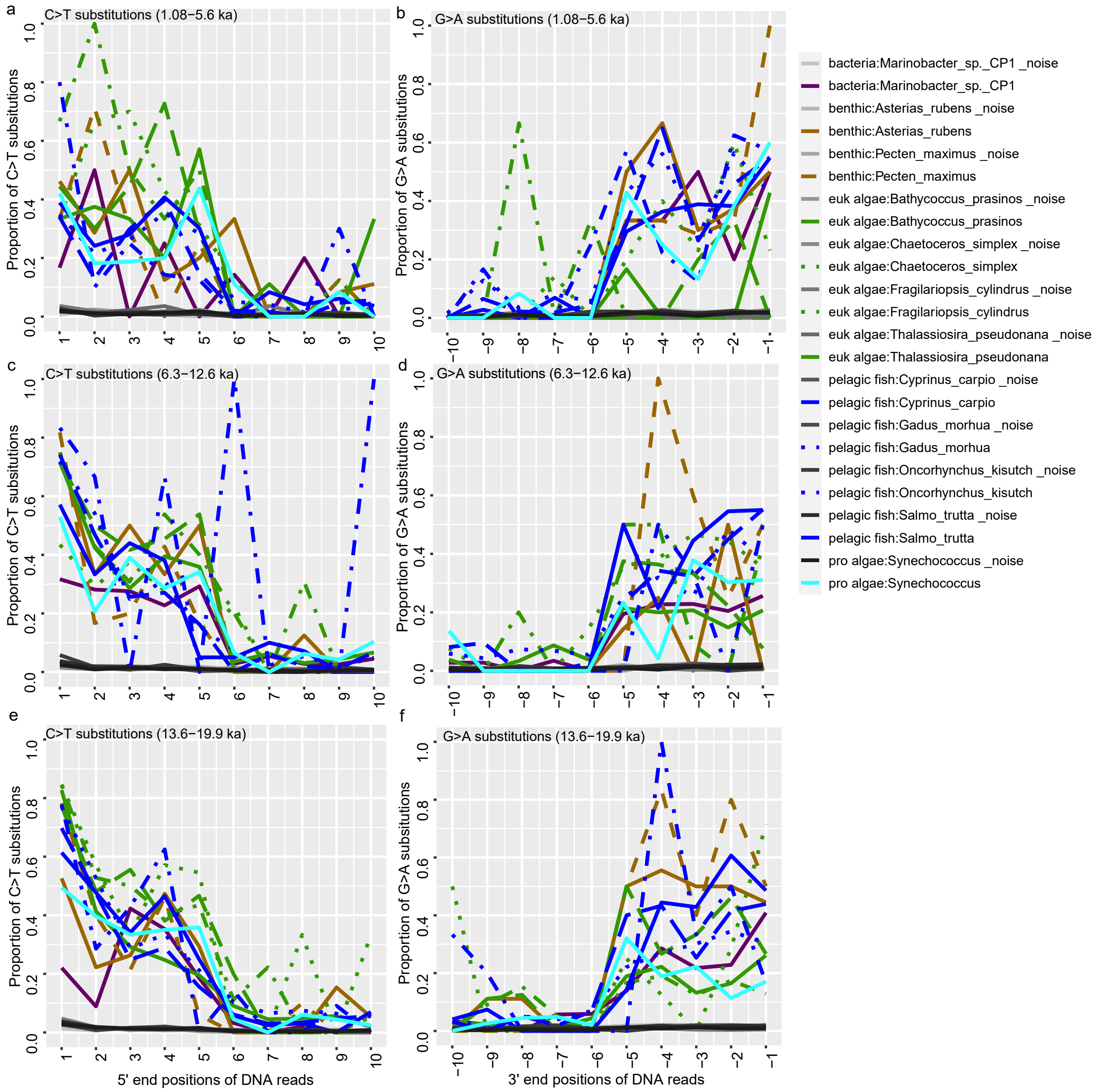

Supplementary Figure 6. Damage profiles of a-b: dataset1 (1.08–5.6 cal kyr BP), c-d: dataset2 (6.3–12.6 cal kyr BP), and e-f: dataset3 (13.6–19.9 cal kyr BP). C>T substitutions in 5' direction and G>A substitutions in 3' are given for each taxon analyzed. Non-C>T substitutions in 5' direction and non-G>A substitutions in 3' direction are given to estimate the noise (gray color). The general color code for benthic taxa (*Asterias rubens* and *Pecten maximus*) is brown shades, eukaryotic algae (euk algae) are green shades, pelagic fish are blue shades, and bacteria are purple. Overall, the profiles show increasing accumulation of damage over the three binned timeframes for pelagic and benthic families.

Supplementary note 5: Signal validation of Salmonidae

Is the signal of Salmonidae versus other fish influenced by its high representation in the NCBI database?

The strength of kraken2 addresses exactly this point. kraken2 connects minimizers (short substrings used for binning of k-mers) with the lowest common ancestor (LCA) taxa. When 2 distinct k-mers from distantly related genomes share the same minimizer, this can either lead to higher LCA values (in case both genomes are part of the database) or incorrect assignments (if one of the genomes is missing). However, the latter problem is stronger on species and genus level than on higher level classifications like family level, especially as our focal families are represented by at least one reference genome. Our method is also less prone to bias by taxa with a high representation in datasets than a BLAST-based approach. BLAST returns the n-best hits, but a model species can dominate the list of hits and a more closely related species with a better hit might be missed if it comes further down in the database and the threshold of hits has been reached.

We assessed the state of the database and tested the signal of Salmonidae versus that of other fish families (here the most important families of the Western Bering Sea food web: Gadidae, Clupeidae, Pleuronectidae). These families are covered by several reference genomes across several genera (Supplementary Table 2). Furthermore, almost all the corresponding genera from our study area are represented in the database.

One important group that is present in our data, but missing in our correlation analyses, due to very few read counts is the family Pleuronectidae, which contains ground fish such as the Pacific halibut (*Hippoglossus stenolepis*; genome available), Arrowtooth flounder (*Atheresthes stomas*; no references in NCBI) and Greenland turbot (*Reinhardtius hippoglossoides*; genome available). The proportion of Pleuronectidae minimizers (0.12%) among Actinopteri is only slightly lower than the proportion of minimizers for Clupeidae (0.15%). Hence other factors than a reference database bias could be responsible for the lower number of read counts assigned to Pleuronectidae, such as lower past abundance compared to Salmonidae and Clupeidae.

Salmonidae have many more unique minimizers in the database than the other fish families (5.21% of Actinopteri minimizers)

Supplementary Table 2. Read counts for planktic, pelagic, and benthic taxonomic groups before resampling.

Family	Genera in Family (FishBase)	Genera represented in NCBI db	Genome	Assemblies	mitochondrial reference genomes	nucleotide sequences	Unique minimizers	% of Actinopteri minimizers
**Salmonidae**	**11**	**11**	**15**	**50**	**56**	**3616430**	**2005295682**	**5.21%**
Coregonus sp.			1					
Salvelinus			1					
Hucho hucho			1					
Salmo trutta			1		1			
Oncorhynchus keta			1		1			
Oncorhynchus gorbuscha			1		1			
Oncorhynchus nerka			1		1			
Oncorhynchus tshawytscha			1		1			
Oncorhynchus kisutch			1		1			
Salvelinus namaycush			1		1			
Coregonus clupeaformis			1		1			
Thymallus thymallus			1		1			
Coregonus lavaretus			1					
Salmo salar			1					
Oncorhynchus mykiss			1		1			
**Gadidae**	**11**	**11**	**9**	**15**	**5**	**382120**	**395536200**	**1.03%**
Gadiculus argenteus			1					
Trisopterus minutus			1					
Gadus chalcogrammus			1					
Pollachius virens			1					
Arctogadus glacialis			1					
Boreogadus saida			1					
Merlangius merlangus			1					
Melanogrammus aeglefinus			1					
Gadus morhua			1		1			
Gadus macrocelphalus					1			
Eleginus gracilis					1			
Micromesistius poutassou					1			
Pollachius pollachius					1			
**Clupeidae**	**52**	**48**	**6**	**21**	**58**	**258014**	**59088162**	**0.15%**
Limnothrissa miodon			1					
Clupea harengus			1		1			
Tenualosa ilisha			1					
Alosa sapidissima			1		1			
Alosa alosa			1		1			
Sardina pilchardus			1					
**Pleuronectidae**	**26**	**29**	**6**	**12**	**8**	**143210**	**44633306**	**0.12%**
Pseudopleuronectes yokohamae			1					
Platichthys stellatus			1					
Hippoglossus stenolepis			1					
Hippoglossus hippoglossus			1					
Reinhardtius hippoglossoides			1					
Verasper variegatus			1					

Could correlations be influenced by a reference database bias of closely related taxa?

To show whether correlations could be influenced by a reference database bias, we performed a test based on the group of fish, which have a sufficiently large number of read counts. The most expected fish families based on their role in the modern western Bering Sea food web are the Salmonidae, Clupeidae, and Gadidae[4]. Our database analysis shows that these are well represented through reference genomes of several genera (Supplementary Table 2).

The analysis shows that for Clupeidae and Gadidae, only genera from the study area determine the signal on family level (Supplementary Fig. 7b,c). Among Clupdeidae, 48 genera are represented in the database and 99.3% (2,877counts) of the reads are assigned to the genus *Clupea*, while the rest (6 counts) are assigned to 5 other genera (Supplementary Fig. 7b).

Among Gadidae, our reads were assigned to 8 of the 12 genera represented in the database. All of them except for *Micromesistius* (2 counts assigned) are present in the Bering Sea or the Pacific Arctic. The majority of reads were assigned to the genus *Gadus* (22,411counts), which is driving the trends on family level. Notable, but few assignments were found for the genus *Boreogadus* (Polar cod; 107counts). Today, Polar cod is a keystone species of the high-Arctic with a strong linkage to sea ice[5], which supports their presence only during the late glacial in our samples.

The Bering Sea, and the Kamchatka area in particular, is a diversity hotspot for Salmonidae and provides spawning grounds for the genera *Oncorhynchus*, *Coregonus* and *Salvelinus*, which are all represented in our data on genus level. Of the overall 11 genera represented in the database, counts were mostly assigned to *Oncorhynchus* (2,648 counts), *Salvelinus* (613 counts), *Coregonus* (17,849 counts), and *Salmo* (18,090 counts), while only 6 counts were assigned to *Thymallus* and 2 to *Hucho*, which do not occur in the Bering Sea. The high assignment of reads to the genus *Salmo* could be a result of its high representation in the database or potential gaps in assemblies of other Salmonidae. This suggests that analyzing our data on family level is more appropriate than on lower taxonomic levels.

In summary, our data suggest that if a family is represented by a genome, kraken2 can manage the taxonomic assignment on this level. Therefore, we are confident that assignments to these families are valid.

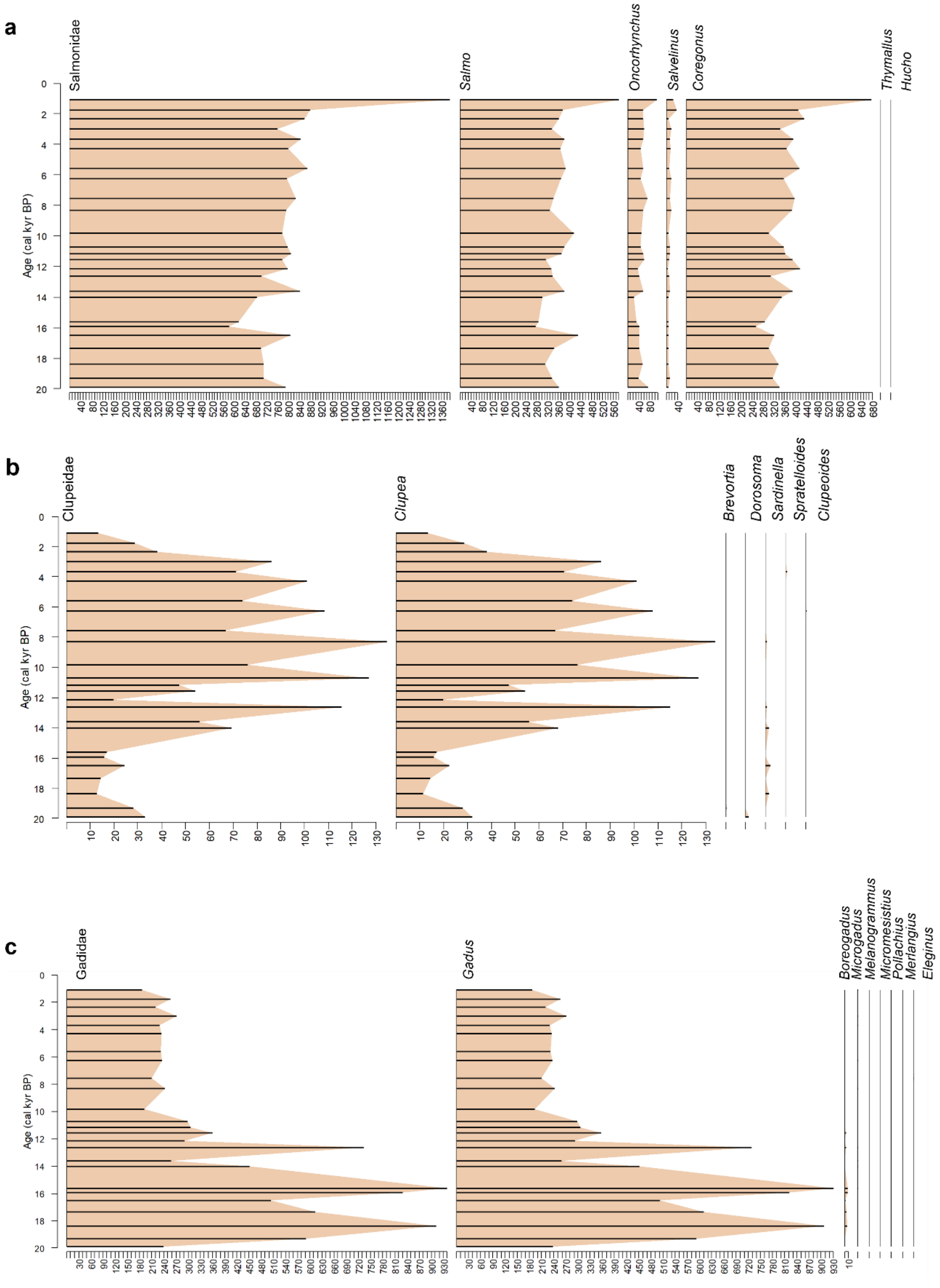

Supplementary Figure 7. Temporal signal of focal fish taxa at family and genus level: (a) Salmonidae, (b) Clupeidae, (c) Gadidae.

Supplementary note 6: Test for temporal autocorrelation

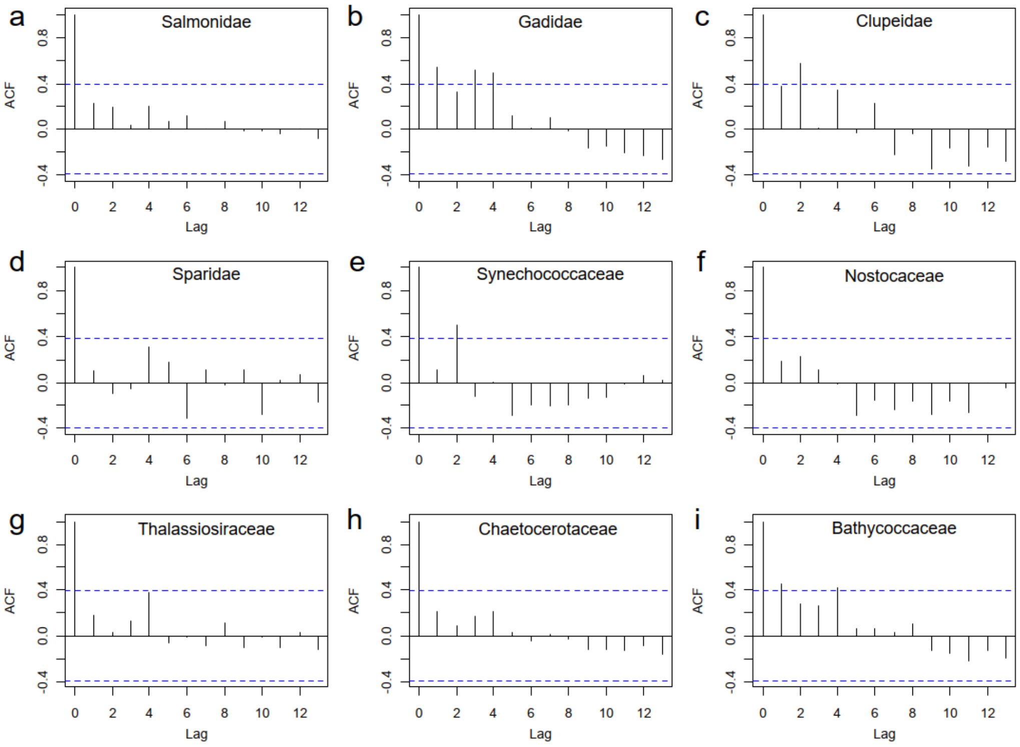

Supplementary Figure 8. Temporal autocorrelation plots of some of the most abundant pelagic taxa among (a-d) fish, (e-f) cyanobacteria, (g-h) diatoms, and (i) chlorophytes suggest that downcore temporal autocorrelation between samples is not affecting the correlation network. The lag 0 autocorrelation is fixed at 1.

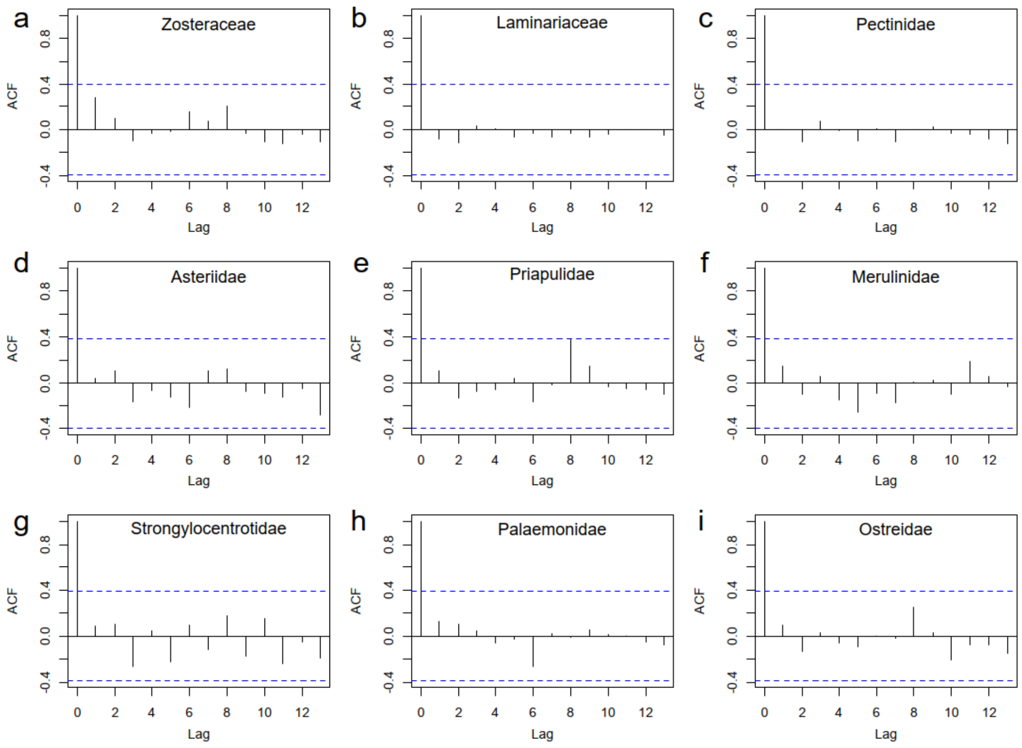

Supplementary Figure 9. Temporal autocorrelation plots of some of the most abundant benthic taxa among (a, b) macrophytes, (b, i) molluscs, (c, g) echinoderms, (e) annelids, (f) cnidarians, and (h) crustaceans suggest that downcore temporal autocorrelation between samples is negligible. The lag 0 autocorrelation is fixed at 1.

Supplementary note 7: Correlation network analysis

Interpolated values

SST and IP_{25}[6] data were interpolated to our sample ages using R function approx[7]. As for the last 2 samples IP_{25} data could not be extrapolated, we calculated the mean of the last three interpolated values and replaced the missing data based on our knowledge about past sea ice coverage during the time for the study area from diatom microfossil- and biomarker-derived sea ice reconstructions for the LGM[8,9]

Supplementary Table 3. Interpolated values for IP_{25} (seasonal sea ice indicator) and SSTs (late summer/early fall temperature indicator).

Age (cal kyr BP)	IP_{25} (µg g^{-1} sediment)	$SST_{UK'37}$ (°C)
1.08	0.002963148	10.62408724
1.75	0.002626213	10.64911873
2.36	0.002154964	11.05654296
2.99	0.001993602	10.63779093
3.66	0.003891224	10.34516472
4.31	0.002803875	9.96946128
5.6	0.004077585	10.34702008
6.26	0.002729124	9.783810255
7.56	0.002782423	10.06475664
8.29	0.003998239	10.46982775
9.84	0.002321933	10.86202396
10.73	0.002810701	10.57618842
11.17	0.006094	10.3278189
11.56	0.004425627	10.04186022
12.13	0.028385708	7.14345425
12.61	0.020262972	7.313923674
13.62	0.004270033	9.318954252
14.03	0.005828588	8.921210969
15.6	0.022091014	6.70318985
15.91	0.018019808	6.59466641
16.5	0.029575612	6.992239568
17.34	0.01472526	5.695324051
18.39	0.018176429	7.306682467
19.3	0.018176429	7.27120261
19.9	0.018176429	7.466771735

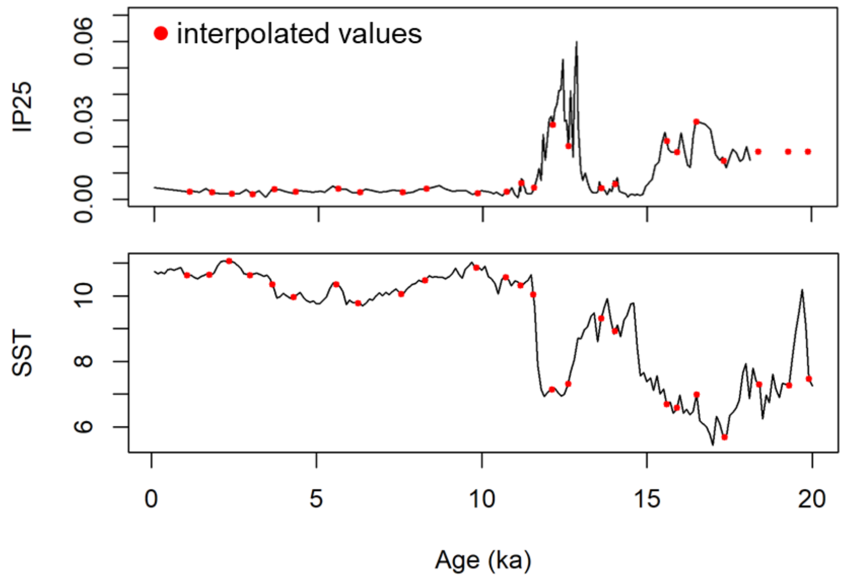

Supplementary Figure 10. Interpolated and extrapolated values for the environmental variables IP_{25} (µg g^{-1} sediment) and $SST_{Uk'37}$ (°C).

Spearman correlation networks

Supplementary Table 4. Correlations (Spearman's ρ >0.4) and Benjamini-Hochberg adjusted p-values of nodes (family names) with SSTs (late summer/early fall temperature indicator) and IP_{25} (seasonal sea ice indicator) variables.

		**SSTs**		**IP_{25}**	
**Group**	**Family name**	**ρ**	**p-value**	**ρ**	**p-value**
pelagic	Gadidae	-0.7408	0.0043	0.7198	0.0067
pelagic	Oxytrichidae	-0.7387	0.0046	0.7566	0.0028
pelagic	Bacillariaceae	-0.7205	0.0067	0.6860	0.0133
pelagic	Metridinidae	-0.7082	0.0083	0.6221	0.0368
pelagic	Bathycoccaceae	-0.7008	0.0100	0.6913	0.0120
pelagic	Phaeocystaceae	-0.6838	0.0140	0.5881	0.0572
pelagic	Ulnariaceae	-0.6409	0.0276	0.4631	0.1864
pelagic	Fragilariaceae	-0.6336	0.0315	0.5328	0.1038
pelagic	Ulvaceae	-0.6229	0.0365	0.6615	0.0208
pelagic	Naviculaceae	-0.6146	0.0414	0.5096	0.1274
pelagic	Suessiaceae	-0.5441	0.0929	0.5985	0.0517
pelagic	Pycnococcaceae	-0.5302	0.1053		
pelagic	Amphipleuraceae	-0.5302	0.1053		
pelagic	Attheyaceae	-0.5292	0.1060	0.4781	0.1676
pelagic	Holocentridae	-0.5123	0.1242		
pelagic	Thalassiosiraceae	-0.5123	0.1242		
pelagic	Stephanodiscaceae	-0.4977	0.1429		
pelagic	Chroomonadaceae	-0.4849	0.1600		
pelagic	Cymatosiraceae	-0.4688	0.1796		
pelagic	Toxariaceae	-0.4528	0.1983		
pelagic	Noelaerhabdaceae	0.4538	0.1967		
pelagic	Selenastraceae	0.4544	0.1961	-0.5087	0.1289
pelagic	Eucalanidae	0.4590	0.1903		
pelagic	Serranidae	0.4608	0.1891		
pelagic	Gloeobacteraceae	0.4654	0.1841	-0.5327	0.1038
pelagic	Merismopediaceae	0.4669	0.1821		
pelagic	Bovichtidae	0.4800	0.1655		
pelagic	Clupeidae	0.4815	0.1629	-0.4873	0.1573
pelagic	Fonticulaceae	0.4924	0.1506	-0.4581	0.1914
pelagic	Pleuronectidae	0.5131	0.1238	-0.4742	0.1716
pelagic	Salpingoecidae	0.5205	0.1145		
pelagic	Desmidiaceae	0.5258	0.1086	-0.4884	0.1563
pelagic	Rhincodontidae	0.5269	0.1076		
pelagic	Nephroselmidaceae	0.5356	0.1012	-0.5652	0.0760
pelagic	Chlorobiaceae	0.5577	0.0814	-0.5874	0.0580
pelagic	Microcystaceae	0.5777	0.0659	-0.5681	0.0732

pelagic	Sciaenidae	0.5838	0.0605	-0.5127	0.1242
pelagic	Klebsormidiaceae	0.5954	0.0534	-0.6377	0.0293
pelagic	Blenniidae	0.6085	0.0449		
pelagic	Sebastidae	0.6178	0.0398	-0.5859	0.0592
pelagic	Salmonidae	0.6185	0.0393	-0.4550	0.1956
pelagic	Chlorellaceae	0.6328	0.0318	-0.5120	0.1247
pelagic	Chaetocerotaceae	0.6477	0.0252	-0.7552	0.0028
pelagic	Hyellaceae	0.6483	0.0251	-0.4681	0.1803
pelagic	Syngnathidae	0.6577	0.0218	-0.4796	0.1658
pelagic	Roseiflexaceae	0.6769	0.0158	-0.5727	0.0699
pelagic	Mustelidae	0.7346	0.0047	-0.6936	0.0117
pelagic	Acanthocerataceae			-0.5940	0.0541
pelagic	Trebouxiaceae			-0.4654	0.1841
pelagic	Chlorococcaceae			-0.4577	0.1920
pelagic	Calanidae			0.4698	0.1775
benthic	Bangiaceae	0.6473	0.1668		
benthic	Zosteraceae	0.7319	0.0265	-0.6854	0.0970

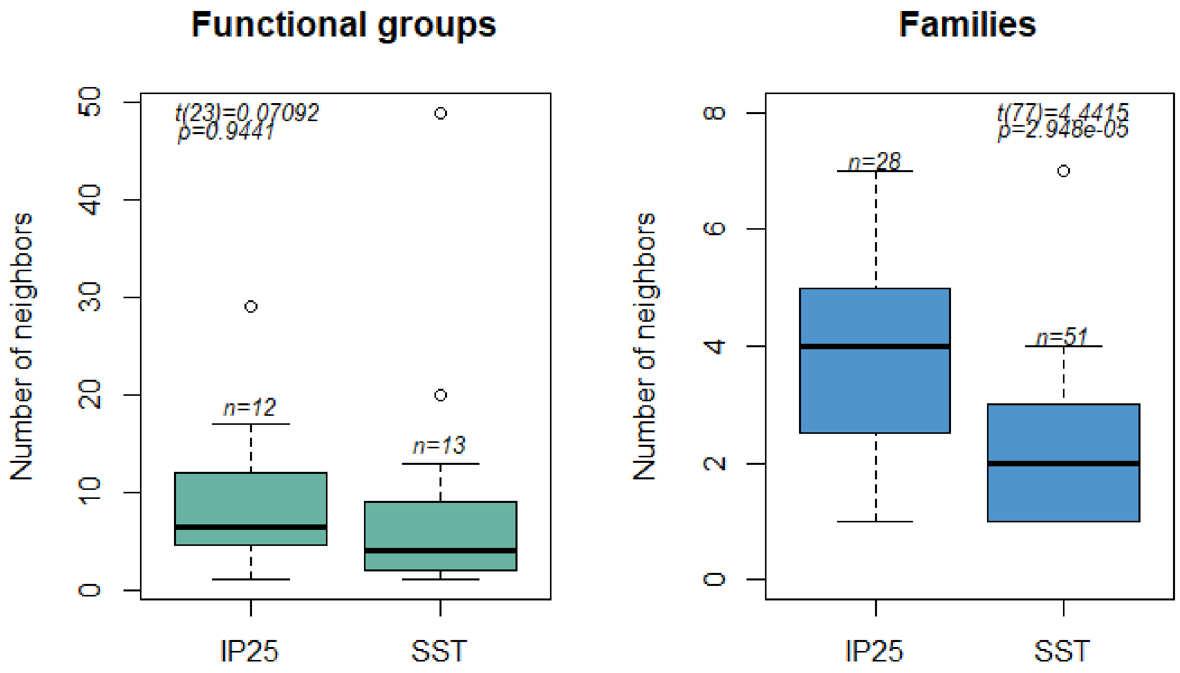

Supplementary Figure 11. Neighbors of IP_{25}- and SST-correlated families. Boxplots showing the number of links to adjacent nodes (neighbors) of IP_{25}- and SST-correlated nodes on the level of functional groups (green) and family (blue). Nodes which are correlated (Spearman's $\rho > 0.4$, adjusted p-value < 0.1) with IP_{25} are significantly connected to more families in comparison to SST-correlated nodes (two sample t-test), but not significantly to more functional groups. The median is represented by the horizontal line inside the box, while lower and upper ends of the box represent the 25th and 75th percentiles, respectively. The upper ends of the whiskers correspond to the smallest and largest values of the 1.5 times interquartile ranges below the 25th and above the 75th percentiles. Outliers are marked as individual circles outside the box.

Sensitivity to zero-inflation

To evaluate the influence of zero inflation on the network, we assessed the distribution of the abundance matrix (Supplementary Fig. 12b), analyzed which role zero-inflated taxa have within the resulting networks (Supplementary Fig. 12a). These analyses have shown that (1) zero inflation is not strongly pronounced in our dataset, (2) the affected taxa have no significant role within the network and (3) that zero-inflation does not have an effect on the creation of the network.

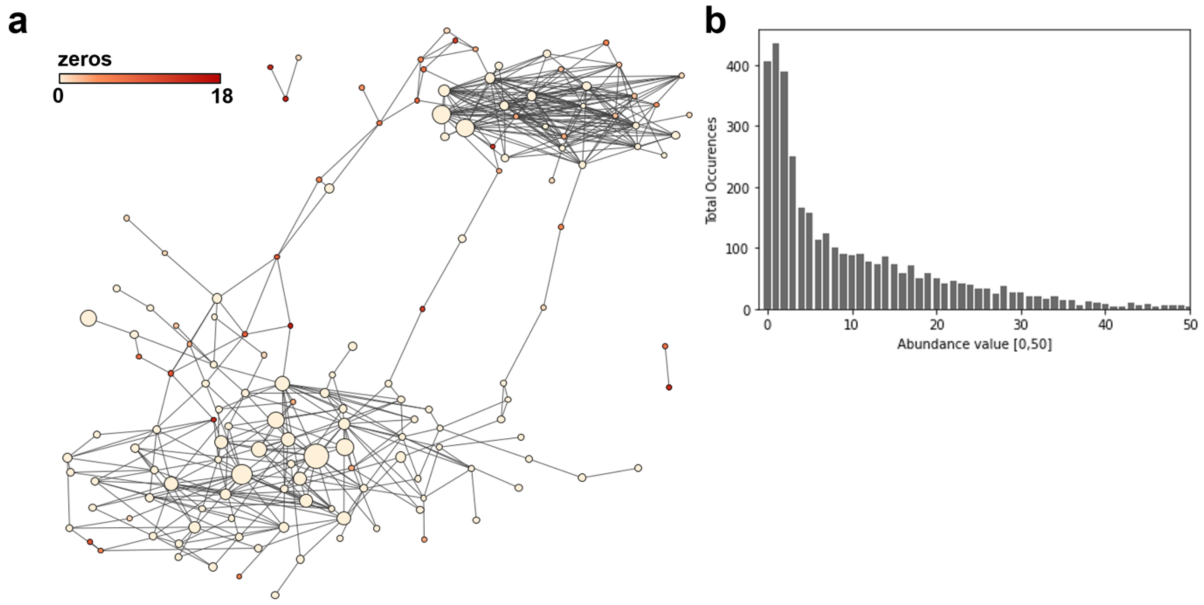

Supplementary Figure 12. (a) Spearman correlation network with nodes colored according to the number of zeros in the pelagic *sed*aDNA time-series. (b) The histogram shows that the occurrences of zeros in the dataset is not extremely inflated. For better representation of zeros in the histogram, only abundance values from 0 to 50 are shown.

Sensitivity to detect non-linear relationships

The Spearman method is a non-linear rank-based correlation, which detects monotonic relationships. This approach to infer a network focuses on the generation of a co-occurrence network, where links between taxa do not necessarily mean a direct relationship or even interaction, but rather similar (with Spearman non-linear) shifts of abundances over time. On this time scale, each sample represents a local snapshot of a distinct state defined by major environmental drivers such as SST or sea ice: identifying actual relationships between the taxa would require a higher resolution of the time series.

We made further efforts to generate networks based on the ecoCopula method which considers environmental covariates as well as mediator taxa. We chose the Jaccard similarity as a simple, intuitive metric for binary data to measure how many edges are present in both networks. Following Tantardini et al.[10], the simplest way of evaluating differences between two adjacency matrices is to compute differences directly, for which several metrics can be used. Since (1) we are not including the edge weights and only need a binary metric (an edge is either present or absent) and (2) shared presences of edges are more informative than shared absences of edges (asymmetric similarity), the Jaccard similarity[11], which divides the intersection of edges by the union of edges, is therefore appropriate. Shared absences might not reflect the similarity at all[12], therefore other indices, such as euclidean distance were omitted. These analyses have shown that the co-occurrence network based on Spearman correlation have a Jaccard Similarity of 0.34 (positive correlations) with networks inferred with ecoCopula. This result shows that the statements of our analysis based on the co-occurrence network remain consistent, given that the ecoCopula network has fewer edges as well as rewiring without a large impact on the network.

Gaussian copula graphical model (ecoCopula network) and comparisons with Spearman network

Pelagic networks

Both the Spearman- (Fig. 2 in main manuscript) and the ecoCopula-network (Supplementary Fig. 13a) have a significant overlap of edges (29% of all and 34% of only positive edges are similar) and are significantly different from the randomized null-model (Supplementary Fig. 13b).

The edge density of the ecoCopula network depends on the shrinking parameter lambda for which an optimal value was determined empirically. Lambda can range from 0 to 1 and we computed ecoCopula networks for each lambda value between 0.1 and 1.0 in 0.1 increments. We show that for increasing lambda values the network becomes less dense (Supplementary Fig. 13b). For each generated ecoCopula network we compared the overlap of edges (all associations, only positive associations, and only negative associations) with the Spearman-network. The highest similarity between the two network approaches was found at a lambda value of 0.51. Furthermore, we could show that the similarity of edges is higher for only positive associations (34%) compared to all (29%) or only negative associations (0.24%). The significance was tested using a null-model which was generated via double-edge swaps on the ecoCopula networks. This was performed ten times for each lambda value between 0.1 and 1.0 (Supplementary Fig. 14b). The network edge density for the ecoCopula network at lambda of 0.51 results in a comparable number of edges to the Spearman network, which is slightly lower due to the removal of associations resulting from correlation with environmental factors via the included covariates IP_{25} and SST in the stacked species regression model of ecoCopula.

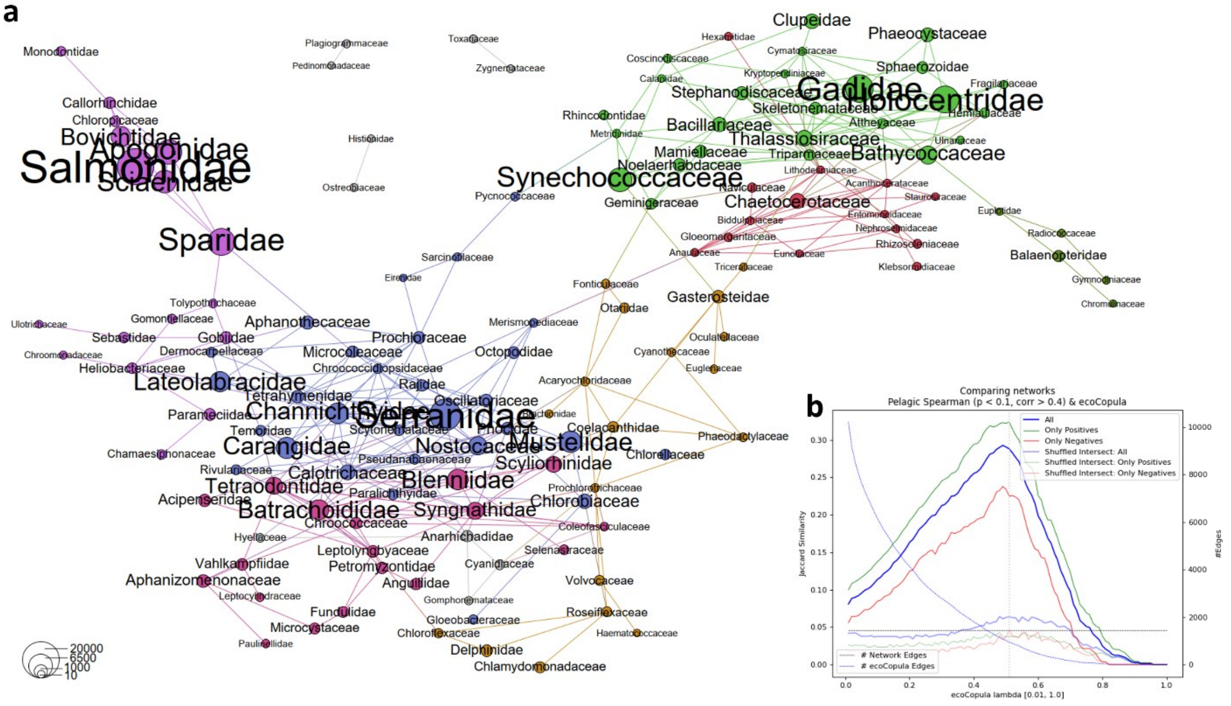

Supplementary Figure 13. (a) Pelagic ecoCopula network of positive associations for lambda of 0.51 with colors highlighting the different modules. (b) Edge overlap (Jaccard similarity) of ecoCopula networks computed for lambda values between 0.1 and 1.0 with the pelagic Spearman correlation-based network. The number of edges in the Spearman network (positive and negative associations) are indicated by the horizontal dotted line while the number of edges decreases with increasing lambda for the ecoCopula networks. The vertical line indicates the optimal lambda (0.51) at which the edge overlap between the two approaches is highest.

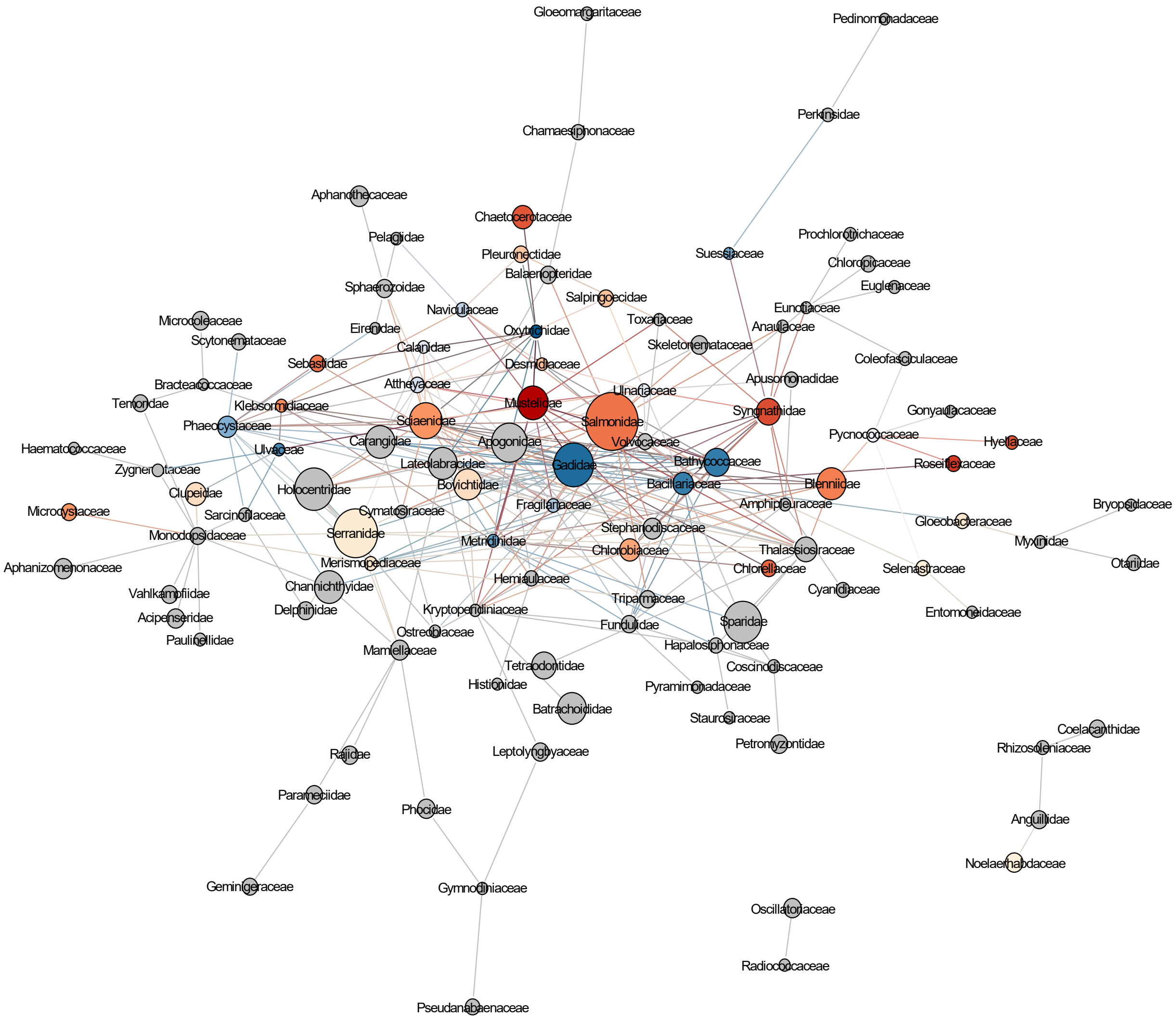

Supplementary Figure 14. Network showing negative associations of the pelagic co-occurrence dataset based on Spearman rank correlation coefficients (> 0.4, adjusted p-value < 0.1). Negative associations are found between families that are positively correlated with IP_{25} (blue nodes) and families that are positively correlated with SSTs (red nodes).

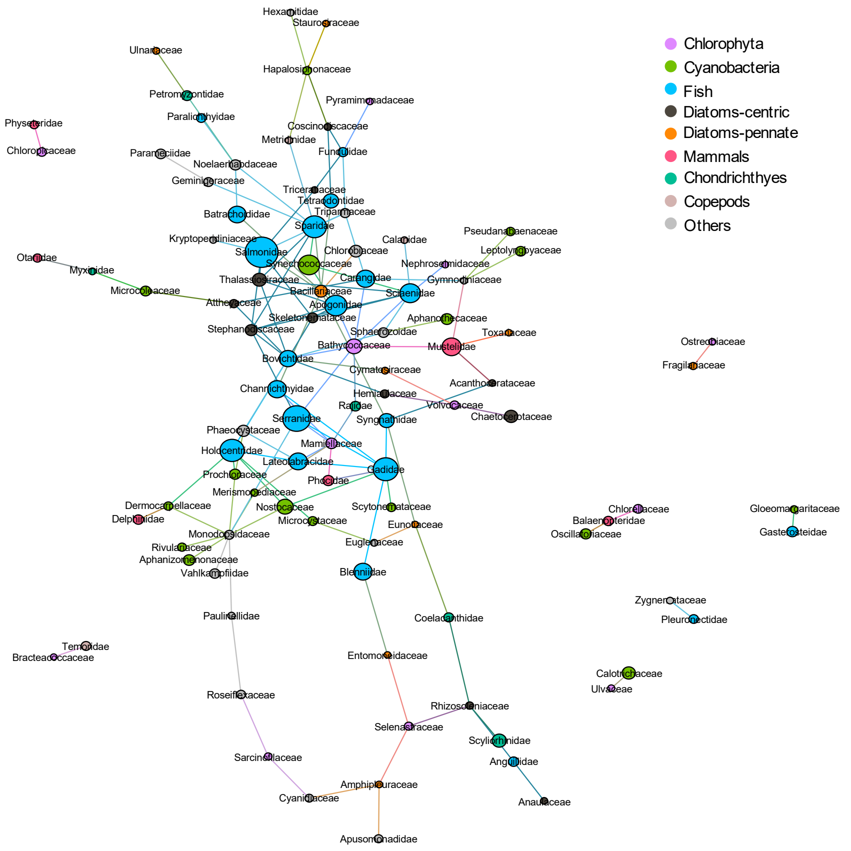

Supplementary Figure 15. Network showing negative associations of the pelagic ecoCopula network with colors representing functional groups.

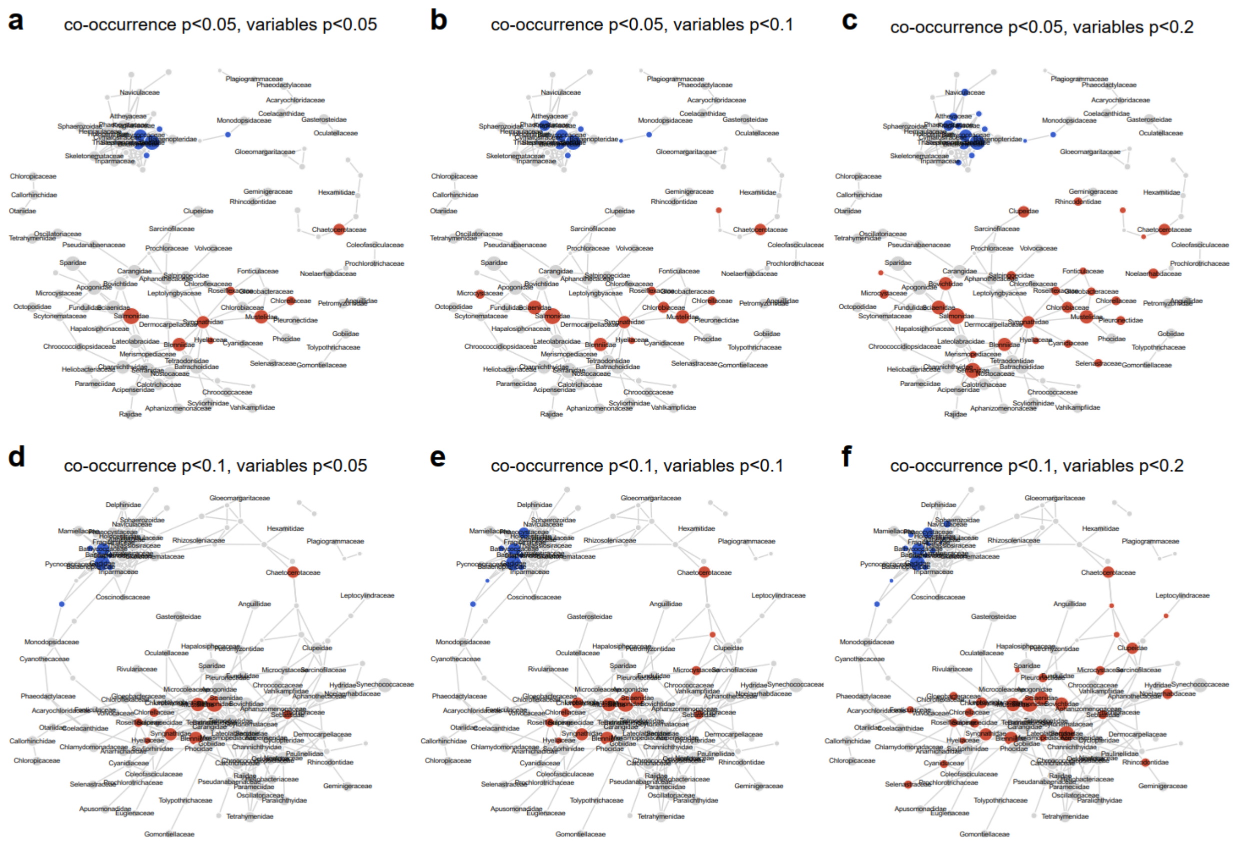

Supplementary Figure 16. Effect of Benjamini-Hochberg adjusted p-value threshold ($p < 0.05$ (a-c) and $p<0.1$ (d-f)) on the network topology. Nodes showing a positive trend (Spearman rank correlations > 0.5 with thresholds of adjusted p-value set to $p<0.05$ (a,d), $p<0.1$ (b,e) and $p< 0.2$(c,f)) with the seasonal sea-ice biomarker IP_{25} are colored blue or with $SST_{UK'37}$ are colored red.

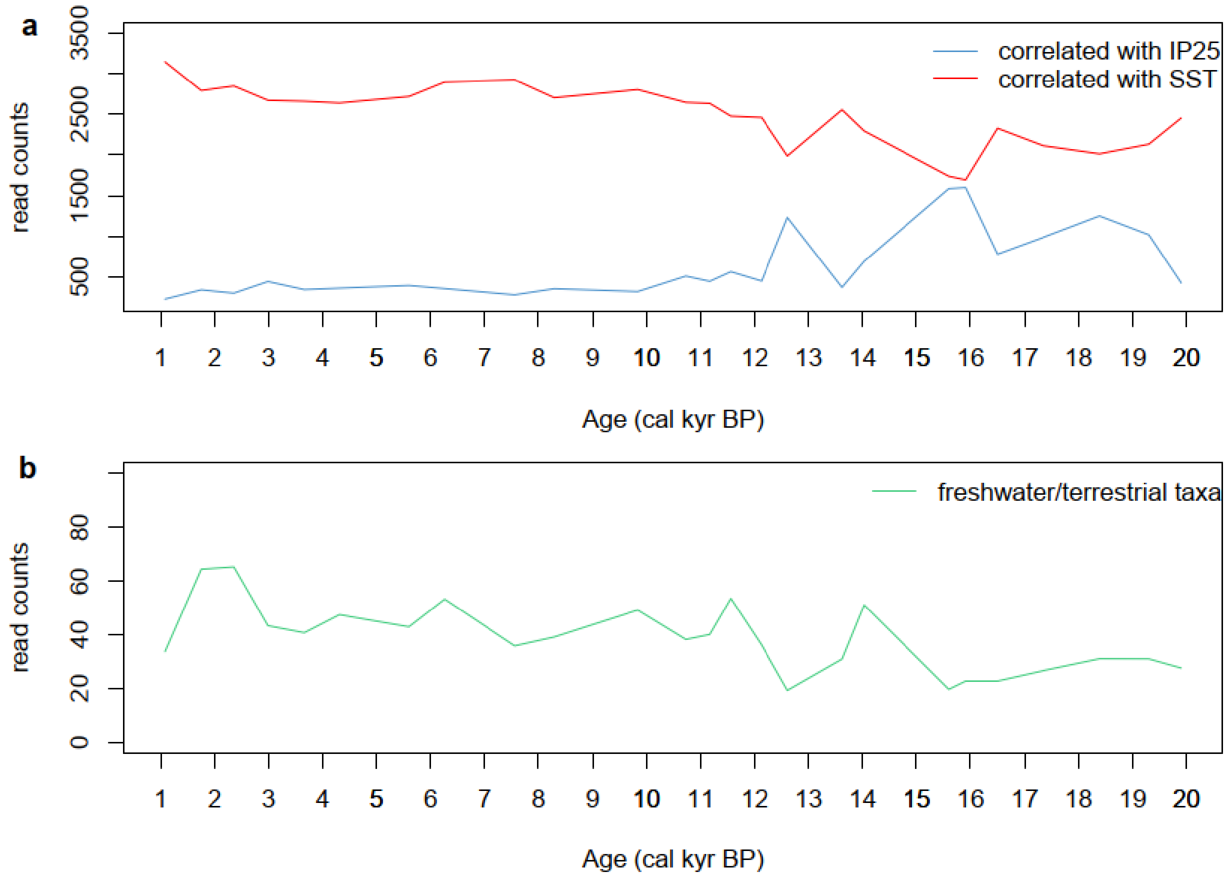

Supplementary Figure 17. Temporal development of read counts assigned to (a) pelagic families correlated with IP_{25} (blue) and $SST_{UK'37}$ (red) and (b) read counts assigned to planktonic families from freshwater or other terrestrial habitats (sum of Acanthocerataceae, Bracteacoccaceae, Chloroflexaceae, Closteriaceae, Cyanophoraceae, Golenkiniaceae, Haematococcaceae, Mesotaeniaceae, Oedogoniaceae, Roseiflexaceae, Sarcinofilaceae, Scotinosphaeraceae, Selenastraceae, Trebouxiaceae, Zygnemataceae), such as hot springs, indicative of terrestrial runoff (green).

Supplementary References

1. Paradis, E. & Schliep, K. ape 5.0: an environment for modern phylogenetics and evolutionary analyses in R. *Bioinformatics* **35**, 526–528 (2019).
2. Hübler, R. *et al.* HOPS: automated detection and authentication of pathogen DNA in archaeological remains. *Genome Biol* **20**, (2019).
3. Huson, D. H. *et al.* MEGAN Community Edition - Interactive Exploration and Analysis of Large-Scale Microbiome Sequencing Data. *PLOS Computational Biology* **12**, e1004957 (2016).
4. Aydin, K. & Mueter, F. The Bering Sea—A dynamic food web perspective. *Deep Sea Research Part II: Topical Studies in Oceanography* **54**, 2501–2525 (2007).
5. Kohlbach, D. *et al.* Strong linkage of polar cod (*Boreogadus saida*) to sea ice algae-produced carbon: Evidence from stomach content, fatty acid and stable isotope analyses. *Progress in Oceanography* **152**, 62–74 (2017).
6. Méheust, M., Stein, R., Fahl, K., Max, L. & Riethdorf, J.-R. High-resolution IP25 of sediment core SO201-2-12. *In supplement to: Méheust, M et al. (2015): High-resolution IP25-based reconstruction of sea-ice variability in the western North Pacific and Bering Sea during the past 18,000 years. Geo-Marine Letters, https://doi.org/10.1007/s00367-015-0432-4* (2015) doi:https://doi.org/10.1594/PANGAEA.855451.
7. R Core Team. *R: A language and environment for statistical computing*. (R Foundation for Statistical Computing, 2018).
8. Maier, E. *et al.* North Pacific freshwater events linked to changes in glacial ocean circulation. *Nature* **559**, 241–245 (2018).
9. Matul, A. G. Probable limits of sea ice extent in the northwestern Subarctic Pacific during the last glacial maximum. *Oceanology* **57**, 700–706 (2017).
10. Tantardini, M., Ieva, F., Tajoli, L. & Piccardi, C. Comparing methods for comparing networks. *Sci Rep* **9**, 17557 (2019).
11. Jaccard, P. Distribution de la flore alpine dans le Bassin des Dranses et dans quelques regions voisines. *Bull Soc Vaudoise Sci Nat* 241–272 (1901).
12. Brusco, M., Cradit, J. D. & Steinley, D. A comparison of 71 binary similarity coefficients: The effect of base rates. *PLOS ONE* **16**, e0247751 (2021).
