# Supplementary material for: Marine ecosystem shifts with deglacial sea-ice loss inferred from ancient DNA shotgun sequencing
Source: Nat Commun. 2023 Mar 24;14:1650. doi: 10.1038/s41467-023-36845-x (PMC10039020; doi:10.1038/s41467-023-36845-x)
Supplement: Supplementary file 3 — Description of Additional Supplementary Files [file 41467_2023_36845_MOESM3_ESM.pdf]

File name: Supplementary Data 1

Description: Processing steps of sequencing data showing how many read counts are retained at each step for each sample, extraction blank (EB) or library blank (LB).

File name: Supplementary Data 2

Description: This file contains the list of families, their grouping into habitat (pelagic/benthic) and trophic status (phototrophic/heterotrophic), the taxonomic group to which the family belongs, the resampled number of read counts used for the formal analysis, links (edges) in the pelagic network, and Spearman correlation coefficients ($\rho>0.2$) and Benjamini-Hochberg adjusted p-values between families and environmental variables (SSTs and IP_{25}).
