# Supplementary material for: Marine ecosystem shifts with deglacial sea-ice loss inferred from ancient DNA shotgun sequencing
Source: Nat Commun. 2023 Mar 24;14:1650. doi: 10.1038/s41467-023-36845-x (PMC10039020; doi:10.1038/s41467-023-36845-x)
Supplement: Supplementary file 5 — Reporting Summary [file 41467_2023_36845_MOESM5_ESM.pdf]

nature portfolio

Corresponding author(s): Ulrike Herzschuh

Last updated by author(s): Dec 23, 2022

Reporting Summary

Nature Portfolio wishes to improve the reproducibility of the work that we publish. This form provides structure for consistency and transparency in reporting. For further information on Nature Portfolio policies, see our Editorial Policies and the Editorial Policy Checklist.

Statistics

For all statistical analyses, confirm that the following items are present in the figure legend, table legend, main text, or Methods section.

n/a	Confirmed	
☐	☒	The exact sample size (*n*) for each experimental group/condition, given as a discrete number and unit of measurement
☒	☐	A statement on whether measurements were taken from distinct samples or whether the same sample was measured repeatedly
☐	☒	The statistical test(s) used AND whether they are one- or two-sided<br>*Only common tests should be described solely by name; describe more complex techniques in the Methods section.*
☐	☒	A description of all covariates tested
☐	☒	A description of any assumptions or corrections, such as tests of normality and adjustment for multiple comparisons
☐	☒	A full description of the statistical parameters including central tendency (e.g. means) or other basic estimates (e.g. regression coefficient) AND variation (e.g. standard deviation) or associated estimates of uncertainty (e.g. confidence intervals)
☐	☒	For null hypothesis testing, the test statistic (e.g. *F*, *t*, *r*) with confidence intervals, effect sizes, degrees of freedom and *P* value noted<br>*Give P values as exact values whenever suitable.*
☒	☐	For Bayesian analysis, information on the choice of priors and Markov chain Monte Carlo settings
☒	☐	For hierarchical and complex designs, identification of the appropriate level for tests and full reporting of outcomes
☐	☒	Estimates of effect sizes (e.g. Cohen's *d*, Pearson's *r*), indicating how they were calculated

Our web collection on statistics for biologists contains articles on many of the points above.

Software and code

Policy information about availability of computer code

Data collection	The IP25 and SST datasets are open access, cited in the text, and were retrieved from PANGAEA or the supplementary information of the corresponding paper:<br>- IP25: https://doi.org/10.1594/PANGAEA.855451<br>- SST: Data File S1; https://advances.sciencemag.org/content/6/9/eaay2915/tab-figures-data
Data analysis	The data analysis is based on open access software.<br>Sequence processing and taxonomic classifications:<br>quality check: FastQC v. 0.11.9, duplicate removal: FastUniq v. 1.1/clumpify within BBMap v. 38.87, trimming and merging of reads: Fastp v. 0.20.0, taxonomic classification: Kraken2 v. 2.0.8-beta<br>Damage pattern analysis: HOPS v. 0.34<br><br>Statistical analysis and network generation were performed in R v. 4.0.3:<br>pairwise Spearman rank correlation coefficients: package psych v. 2.0.12<br>network computation and graph: package igraph v. 1.2.6, ecoCopula v. 1.0.2<br>interpolation of SST and IP25 data to our sample depths: approx from package stats v. 4.0.3<br>neighboring nodes:igraph v. 1.2.6, stats v. 4.0.3<br>stratigraphic diagram: package rioja v. 0.9-26 , CONISS dendrogram: package vegan v. 2.5-7

For manuscripts utilizing custom algorithms or software that are central to the research but not yet described in published literature, software must be made available to editors and reviewers. We strongly encourage code deposition in a community repository (e.g. GitHub). See the Nature Portfolio guidelines for submitting code & software for further information.

Data

Policy information about availability of data

All manuscripts must include a data availability statement. This statement should provide the following information, where applicable:

- Accession codes, unique identifiers, or web links for publicly available datasets
- A description of any restrictions on data availability
- For clinical datasets or third party data, please ensure that the statement adheres to our policy

The sequencing data generated in this study have been deposited at the European Nucleotide Archive (ENA) under Bioproject number PRJEB46821 [https://www.ebi.ac.uk/ena/browser/view/PRJEB46821]. The metadata and taxonomic count data on family level used in this study are available at PANGAEA [https://doi.org/10.1594/PANGAEA.934664].

Field-specific reporting

Please select the one below that is the best fit for your research. If you are not sure, read the appropriate sections before making your selection.

☐ Life sciences ☐ Behavioural & social sciences ☒ Ecological, evolutionary & environmental sciences

For a reference copy of the document with all sections, see nature.com/documents/nr-reporting-summary-flat.pdf

Ecological, evolutionary & environmental sciences study design

All studies must disclose on these points even when the disclosure is negative.

Study description	In this study we use metagenomic shotgun sequencing of sedimentary ancient DNA to explore pelagic and benthic ecosystem changes off Kamchatka (53.993°N, 162.37°E; 2,173 m depth) in the course of sea-ice variability over the past ~20,000 years. We investigated changes in the composition of pelagic and benthic families over this time-frame and computed correlation networks for each of the two groups based on positive, pairwise Spearman rank correlations (rho > 0.4). We then identified nodes in the networks that are positively correlated with the seasonal sea ice proxy (IP25) or late summer/early fall sea surface temperatures (SSTUK'37) and assessed which functional groups are more prevalent in either the seasonal sea-ice ecosystem or a warmer, ice-free ecosystem.
Research sample	We took a total of 25 samples from the marine sediment core SO201-2-12KL and 5 negative controls (pooled extraction blanks and library preparation blanks) were treated as samples during the whole process of sequence classification and subsequent filtering steps to monitor contamination.
Sampling strategy	The samples were taken at a mean temporal resolution of ~780 years, thereby ensuring that each millenium is represented by at least one and each climatic phase by at least 2 samples.
Data collection	The IP25 and SST datasets are open access, cited in the text, and were retrieved from PANGAEA or the supplementary information of the corresponding paper:<br>- IP25: https://doi.org/10.1594/PANGAEA.855451<br>- SST: Data File S1; https://advances.sciencemag.org/content/6/9/eaay2915/tab-figures-data<br><br>Data used to create the map (Supplementary Figure 1) are cited in the caption:<br>- Subarctic North Pacific and median sea-ice extent for March, 1981–2010: Fetterer, F., Knowles, K., Meier, W. N., Savoie, M. & Windnagel, A. K. Sea Ice Index, Version 3. Monthly and daily GIS compatible shapefiles of median ice extent. National Snow & Ice Data Center (2017) doi:10.7265/n5k072f8.<br>- Modern mean January and mean June chlorophyll-a concentrations from 2020: E.U. Copernicus Marine Service Information; https://resources.marine.copernicus.eu/?option=com_csw&view=details&product_id=GLOBAL_ANALYSIS_FORECAST_BIO_001_028
Timing and spatial scale	The samples were taken at a mean temporal resolution of ~780 years, thereby ensuring that each millenium is represented by at least one and each climatic phase by at least 2 samples. The spatial scale is local to regional as one sediment core was taken, but the site is influenced by two large ocean currents (East Kamchatka current transports water masses from the Bering Sea and Alaskan Stream transports Pacific water masses along the Aleutian Arc), the western subarctic gyre, and terrestrial runoff.
Data exclusions	One sample (11.1 cal kyr BP) was excluded from the anaylsis, because it contained only a small fraction of sequences compared to the other samples. As we used a resampling of reads to compare the samples on an equal amount of sequences, this sample had by far the fewest number of taxonomically classified sequences and thus biased the resampling of the data. We therefore sequenced a sample slightly older (11.17 cal kyr BP).<br>We kept only families belonging to phototrophic bacteria, photo- and heterotrophic protists and Metazoa of likely regional and aquatic origin. Among protists, we excluded parasites, among fish we kept only those which occur in the subarctic North Pacific and the Bering Sea based on FishBase (https://fishbase.mnhn.fr/home.htm), and among Metazoa we excluded Streptophyta, Insecta, Fungi, and terrestrial Chelicerata. Furthermore, we kept only families that occurred in at least 3 samples with at least 10 counts.
Reproducibility	Only a part of the prepared sequencing libraries were sequenced and the remainders are frozen at the Alfred Wegener Institute, Potsdam.

Randomization	After taxonomic classification the classified sequences were grouped into pelagic and benthic organisms based on their habitat information.
Blinding	Blinding was not relevant for this study.

Did the study involve field work? ☒ Yes ☐ No

Field work, collection and transport

Field conditions	Field work conditions auch as rainfall or temperature are not relevant for this study. All relevant details are described in the field report:<br>Dullo, W.-C., B. Baranov, and C. van den Bogaard (Eds.) (2009), FS Sonne Fahrtbericht/Cruise Report SO201–2, IFM-GEOMAR Rep. 35, 233 pp., IFM-GEOMAR, Kiel, Germany.
Location	coring site SO201-2-12KL: 53.993°N, 162.37°E, water depth: 2,173 m bsl
Access & import/export	The processed sediment core SO201-2-12KL was obtained during the expedition "KALMAR" in 2009 with the German research vessel Sonne. The expedition was funded by a project grant from the Federal Ministry of Education and Research (BMBF) as well as by a grant from the Russian Foundation for Basic Research (RFFI N05-08-00017, RFFI 07-05-00807-a) and the Russian Academy of Sciences. The Federal Agency for Science and Innovations of Russia granted permission to work within their territorial waters in the frame of scientific-technical Programme (WTZ) between Russia and Germany as agreed on the Protocol of the 10th WTZ - Working Group Meeting 07.02.2006 as project Nr. 19. Efforts to collect samples in a responsible manner are described below under 'Disturbance'.
Disturbance	Our scientific work followed the "Erklärung zu einer verantwortungsvollen Meeresforschung" and the “Code of Conduct for Responsible Marine Research in the Deep Seas and High Seas of the OSPAR Maritime Area”. Acoustic measurements, water column and sediment sampling have been restricted to methods and areas being essential to conduct the research goals. To minimise the impact of acoustic measurements on marine mammals, MMO observations were conducted. We apply the recommendations of the “Mitigation measures for the operation of seismic and hydroacoustic sources with pulsed sound emissions” (issued by the German Research Foundation (DFG)/Federal Ministry of Education and Research (BMBF)) as part of the environmental permitting process for research activities in the High Seas.

Reporting for specific materials, systems and methods

We require information from authors about some types of materials, experimental systems and methods used in many studies. Here, indicate whether each material, system or method listed is relevant to your study. If you are not sure if a list item applies to your research, read the appropriate section before selecting a response.

Materials & experimental systems

n/a	Involved in the study	
☐	☐	Antibodies
☐	☐	Eukaryotic cell lines
☐	☐	Palaeontology and archaeology
☐	☐	Animals and other organisms
☐	☐	Human research participants
☐	☐	Clinical data
☐	☐	Dual use research of concern

Methods

n/a	Involved in the study	
☐	☐	ChIP-seq
☐	☐	Flow cytometry
☐	☐	MRI-based neuroimaging

Antibodies

Antibodies used	*Describe all antibodies used in the study; as applicable, provide supplier name, catalog number, clone name, and lot number.*
Validation	*Describe the validation of each primary antibody for the species and application, noting any validation statements on the manufacturer’s website, relevant citations, antibody profiles in online databases, or data provided in the manuscript.*

Eukaryotic cell lines

Policy information about cell lines

Cell line source(s)	*State the source of each cell line used.*
Authentication	*Describe the authentication procedures for each cell line used OR declare that none of the cell lines used were authenticated.*
Mycoplasma contamination	*Confirm that all cell lines tested negative for mycoplasma contamination OR describe the results of the testing for mycoplasma contamination OR declare that the cell lines were not tested for mycoplasma contamination.*

Commonly misidentified lines (See ICLAC register)	*Name any commonly misidentified cell lines used in the study and provide a rationale for their use.*

Palaeontology and Archaeology

Specimen provenance	*Provide provenance information for specimens and describe permits that were obtained for the work (including the name of the issuing authority, the date of issue, and any identifying information). Permits should encompass collection and, where applicable, export.*
Specimen deposition	*Indicate where the specimens have been deposited to permit free access by other researchers.*
Dating methods	*If new dates are provided, describe how they were obtained (e.g. collection, storage, sample pretreatment and measurement), where they were obtained (i.e. lab name), the calibration program and the protocol for quality assurance OR state that no new dates are provided.*

☐ Tick this box to confirm that the raw and calibrated dates are available in the paper or in Supplementary Information.

Ethics oversight	*Identify the organization(s) that approved or provided guidance on the study protocol, OR state that no ethical approval or guidance was required and explain why not.*

Note that full information on the approval of the study protocol must also be provided in the manuscript.

Animals and other organisms

Policy information about studies involving animals; ARRIVE guidelines recommended for reporting animal research

Laboratory animals	*For laboratory animals, report species, strain, sex and age OR state that the study did not involve laboratory animals.*
Wild animals	*Provide details on animals observed in or captured in the field; report species, sex and age where possible. Describe how animals were caught and transported and what happened to captive animals after the study (if killed, explain why and describe method; if released, say where and when) OR state that the study did not involve wild animals.*
Field-collected samples	*For laboratory work with field-collected samples, describe all relevant parameters such as housing, maintenance, temperature, photoperiod and end-of-experiment protocol OR state that the study did not involve samples collected from the field.*
Ethics oversight	*Identify the organization(s) that approved or provided guidance on the study protocol, OR state that no ethical approval or guidance was required and explain why not.*

Note that full information on the approval of the study protocol must also be provided in the manuscript.

Human research participants

Policy information about studies involving human research participants

Population characteristics	*Describe the covariate-relevant population characteristics of the human research participants (e.g. age, gender, genotypic information, past and current diagnosis and treatment categories). If you filled out the behavioural & social sciences study design questions and have nothing to add here, write "See above."*
Recruitment	*Describe how participants were recruited. Outline any potential self-selection bias or other biases that may be present and how these are likely to impact results.*
Ethics oversight	*Identify the organization(s) that approved the study protocol.*

Note that full information on the approval of the study protocol must also be provided in the manuscript.

Clinical data

Policy information about clinical studies

All manuscripts should comply with the ICMJEguidelines for publication of clinical research and a completedCONSORT checklist must be included with all submissions.

Clinical trial registration	*Provide the trial registration number from ClinicalTrials.gov or an equivalent agency.*
Study protocol	*Note where the full trial protocol can be accessed OR if not available, explain why.*
Data collection	*Describe the settings and locales of data collection, noting the time periods of recruitment and data collection.*
Outcomes	*Describe how you pre-defined primary and secondary outcome measures and how you assessed these measures.*

Dual use research of concern

Policy information about dual use research of concern

Hazards

Could the accidental, deliberate or reckless misuse of agents or technologies generated in the work, or the application of information presented in the manuscript, pose a threat to:

No	Yes	
☒	☐	Public health
☒	☐	National security
☒	☐	Crops and/or livestock
☒	☐	Ecosystems
☒	☐	Any other significant area

Experiments of concern

Does the work involve any of these experiments of concern:

No	Yes	
☒	☐	Demonstrate how to render a vaccine ineffective
☒	☐	Confer resistance to therapeutically useful antibiotics or antiviral agents
☒	☐	Enhance the virulence of a pathogen or render a nonpathogen virulent
☒	☐	Increase transmissibility of a pathogen
☒	☐	Alter the host range of a pathogen
☒	☐	Enable evasion of diagnostic/detection modalities
☒	☐	Enable the weaponization of a biological agent or toxin
☒	☐	Any other potentially harmful combination of experiments and agents

ChIP-seq

Data deposition

☐ Confirm that both raw and final processed data have been deposited in a public database such as GEO.

☐ Confirm that you have deposited or provided access to graph files (e.g. BED files) for the called peaks.

Data access links<br>*May remain private before publication.*	*For "Initial submission" or "Revised version" documents, provide reviewer access links. For your "Final submission" document, provide a link to the deposited data.*
Files in database submission	*Provide a list of all files available in the database submission.*
Genome browser session<br>(e.g. UCSC)	*Provide a link to an anonymized genome browser session for "Initial submission" and "Revised version" documents only, to enable peer review. Write "no longer applicable" for "Final submission" documents.*

Methodology

Replicates	*Describe the experimental replicates, specifying number, type and replicate agreement.*
Sequencing depth	*Describe the sequencing depth for each experiment, providing the total number of reads, uniquely mapped reads, length of reads and whether they were paired- or single-end.*
Antibodies	*Describe the antibodies used for the ChIP-seq experiments; as applicable, provide supplier name, catalog number, clone name, and lot number.*
Peak calling parameters	*Specify the command line program and parameters used for read mapping and peak calling, including the ChIP, control and index files used.*
Data quality	*Describe the methods used to ensure data quality in full detail, including how many peaks are at FDR 5% and above 5-fold enrichment.*
Software	*Describe the software used to collect and analyze the ChIP-seq data. For custom code that has been deposited into a community repository, provide accession details.*

Flow Cytometry

Plots

Confirm that:

- [ ] The axis labels state the marker and fluorochrome used (e.g. CD4-FITC).
- [ ] The axis scales are clearly visible. Include numbers along axes only for bottom left plot of group (a 'group' is an analysis of identical markers).
- [ ] All plots are contour plots with outliers or pseudocolor plots.
- [ ] A numerical value for number of cells or percentage (with statistics) is provided.

Methodology

Sample preparation	*Describe the sample preparation, detailing the biological source of the cells and any tissue processing steps used.*
Instrument	*Identify the instrument used for data collection, specifying make and model number.*
Software	*Describe the software used to collect and analyze the flow cytometry data. For custom code that has been deposited into a community repository, provide accession details.*
Cell population abundance	*Describe the abundance of the relevant cell populations within post-sort fractions, providing details on the purity of the samples and how it was determined.*
Gating strategy	*Describe the gating strategy used for all relevant experiments, specifying the preliminary FSC/SSC gates of the starting cell population, indicating where boundaries between "positive" and "negative" staining cell populations are defined.*

- [ ] Tick this box to confirm that a figure exemplifying the gating strategy is provided in the Supplementary Information.

Magnetic resonance imaging

Experimental design

Design type	*Indicate task or resting state; event-related or block design.*
Design specifications	*Specify the number of blocks, trials or experimental units per session and/or subject, and specify the length of each trial or block (if trials are blocked) and interval between trials.*
Behavioral performance measures	*State number and/or type of variables recorded (e.g. correct button press, response time) and what statistics were used to establish that the subjects were performing the task as expected (e.g. mean, range, and/or standard deviation across subjects).*

Acquisition

Imaging type(s)	*Specify: functional, structural, diffusion, perfusion.*
Field strength	*Specify in Tesla*
Sequence & imaging parameters	*Specify the pulse sequence type (gradient echo, spin echo, etc.), imaging type (EPI, spiral, etc.), field of view, matrix size, slice thickness, orientation and TE/TR/flip angle.*
Area of acquisition	*State whether a whole brain scan was used OR define the area of acquisition, describing how the region was determined.*

Diffusion MRI ☐ Used ☐ Not used

Preprocessing

Preprocessing software	*Provide detail on software version and revision number and on specific parameters (model/functions, brain extraction, segmentation, smoothing kernel size, etc.).*
Normalization	*If data were normalized/standardized, describe the approach(es): specify linear or non-linear and define image types used for transformation OR indicate that data were not normalized and explain rationale for lack of normalization.*
Normalization template	*Describe the template used for normalization/transformation, specifying subject space or group standardized space (e.g. original Talairach, MNI305, ICBM152) OR indicate that the data were not normalized.*
Noise and artifact removal	*Describe your procedure(s) for artifact and structured noise removal, specifying motion parameters, tissue signals and physiological signals (heart rate, respiration).*

Volume censoring | *Define your software and/or method and criteria for volume censoring, and state the extent of such censoring.*

Statistical modeling & inference

Model type and settings | *Specify type (mass univariate, multivariate, RSA, predictive, etc.) and describe essential details of the model at the first and second levels (e.g. fixed, random or mixed effects; drift or auto-correlation).*

Effect(s) tested | *Define precise effect in terms of the task or stimulus conditions instead of psychological concepts and indicate whether ANOVA or factorial designs were used.*

Specify type of analysis: ☐ Whole brain ☐ ROI-based ☐ Both

Statistic type for inference
(See Eklund et al. 2016) | *Specify voxel-wise or cluster-wise and report all relevant parameters for cluster-wise methods.*

Correction | *Describe the type of correction and how it is obtained for multiple comparisons (e.g. FWE, FDR, permutation or Monte Carlo).*

Models & analysis

n/a	Involved in the study
☐	☐ Functional and/or effective connectivity
☐	☐ Graph analysis
☐	☐ Multivariate modeling or predictive analysis

Functional and/or effective connectivity | *Report the measures of dependence used and the model details (e.g. Pearson correlation, partial correlation, mutual information).*

Graph analysis | *Report the dependent variable and connectivity measure, specifying weighted graph or binarized graph, subject- or group-level, and the global and/or node summaries used (e.g. clustering coefficient, efficiency, etc.).*

Multivariate modeling and predictive analysis | *Specify independent variables, features extraction and dimension reduction, model, training and evaluation metrics.*
